# The accuracy of protein models automatically built into cryo-EM maps with *ARP*/*wARP*

**DOI:** 10.1107/S2059798320016332

**Published:** 2021-01-26

**Authors:** Grzegorz Chojnowski, Egor Sobolev, Philipp Heuser, Victor S. Lamzin

**Affiliations:** a European Molecular Biology Laboratory, c/o DESY, Notkestrasse 85, 22607 Hamburg, Germany

**Keywords:** *ARP*/*wARP*, model building, cryo-EM, model accuracy, sequence assignment

## Abstract

Recent developments in cryogenic electron microscopy (cryo-EM) have enabled structural studies of large macromolecular complexes at resolutions previously only attainable using macromolecular crystallography. Although a number of methods can already assist in *de novo* building of models into high-resolution cryo-EM maps, automated and reliable map interpretation remains a challenge. Presented here is a systematic study of the accuracy of models built into cryo-EM maps using *ARP*/*wARP*. It is demonstrated that the local resolution is a good indicator of map interpretability, and for the majority of the test cases *ARP*/*wARP* correctly builds 90% of main-chain fragments in regions where the local resolution is 4.0 Å or better. It is also demonstrated that the coordinate accuracy for models built into cryo-EM maps is comparable to that of X-ray crystallographic models at similar local cryo-EM and crystallographic resolutions. The model accuracy also correlates with the refined atomic displacement parameters.

## Abstract 2

A new module of the *ARP*/*wARP* suite for automated model building into cryo-EM maps is presented.

## 1. Introduction   

Unlike X-ray crystallography, cryogenic electron microscopy (cryo-EM) does not require crystalline specimens, which makes it well suited for studying large, structurally heterogeneous macromolecules that may be reluctant to crystallize (Nogales & Scheres, 2015 ▸). Recent developments in detector technology and advances in data-processing algorithms have enabled cryo-EM maps to be obtained at resolutions only previously attainable using X-ray crystallography (Wlodawer *et al.*, 2017 ▸). However, even a high-resolution map needs to be interpreted in terms of an atomic model to be useful in explaining biological processes (Renaud *et al.*, 2018 ▸). An accurate model can be built manually with the use of computer graphics, for example using *Coot* (Emsley *et al.*, 2010 ▸). This, however, can be time-consuming, error-prone and require expert knowledge when the features of a three-dimensional map need to be interpreted visually. Therefore, objective, robust and automated approaches are urgently required, particularly for the model building of large complexes.

A number of software tools can assist in the *de novo* building of models into cryo-EM maps. These include the traditionally crystallographic methods *ARP*/*wARP* (Langer *et al.*, 2008 ▸), *Buccaneer* (Cowtan, 2006 ▸) and *phenix.map_to_model* (Terwilliger *et al.*, 2018 ▸), as well as the structure-prediction program *Rosetta* (Wang *et al.*, 2015 ▸), which have been adapted for the interpretation of cryo-EM maps. There are also software solutions developed specifically for the interpretation of cryo-EM maps: *EMBuilder* (Zhou *et al.*, 2017 ▸), *MAINMAST* (Terashi & Kihara, 2018 ▸) and *Pathwalker* (Chen *et al.*, 2016 ▸).

In macromolecular crystallography, automated model building plays an important role in the structure-determination process. It considerably reduces human effort, and in difficult cases may provide partial models that enable unambiguous manual completion. Importantly, automated model building is also routinely used to evaluate potential solutions of the phase problem (Keegan *et al.*, 2018 ▸; Panjikar *et al.*, 2009 ▸).

Automated model building has already become applicable to cryo-EM. The recently released *ARP*/*wARP* version 8.0 with a module for the interpretation of cryo-EM maps (https://news.embl.de/lab-matters/arp-warp-8-0-released/) has already provided models of a number of macromolecular structures (Gopalasingam *et al.*, 2019 ▸; Radamaker *et al.*, 2019 ▸). It has also served as a means of evaluating the interpretability of maps obtained using different experimental protocols (Song *et al.*, 2019 ▸). Interestingly, the possibility of using automated model building and refinement for the systematic assessment of map modellability has already been pursued. Mendez and Stagg used *Rosetta* for the *de novo* building of protein model ensembles into cryo-EM maps obtained at different resolutions (Mendez & Stagg, 2018 ▸). They reported that the overall resolution estimates based on the Fourier shell correlation (FSC) curves are not always consistent with the overall modellability of the maps and that in many cases the highest resolution maps are not necessarily the easiest to interpret using the algorithms implemented in *Rosetta*. In another analysis, Herzik and coworkers used *Rosetta* for refinement of the deposited models against the corresponding cryo-EM maps reprocessed at different resolutions (Herzik *et al.*, 2019 ▸). They analysed the differences on a single-residue level between independently refined models and observed that models refined against lower resolution maps have a larger fraction of regions with a high structural divergence of C^α^-atom positions, up to a root-mean-square-deviation (r.m.s.d.) of 4 Å. They also noticed that highly divergent model regions correlate with low local resolution of the map and high atomic displacement parameters (ADPs) of the refined models. In both of the studies mentioned above it was noted that the models automatically built or refined using *Rosetta* may provide an insight into the overall modellability of cryo-EM maps. At the same time, the authors noted that both the refined and *de novo* traced models used in the studies were incorrectly traced in some regions, which may have affected the validity of the conclusions drawn. These studies emphasized the importance of assessing the overall reliability and accuracy of atomic models obtained from the automated interpretation of cryo-EM maps.

Presented here is a systematic assessment of the quality of models built *de novo* and automatically into cryo-EM maps using *ARP*/*wARP*. We evaluate the completeness and correctness of the models as a function of resolution estimates based on the FSC curves and local map resolution calculated using *ResMap* (Kucukelbir *et al.*, 2014 ▸). We also estimate the coordinate errors of the *ARP*/*wARP* models for different local map resolutions and compare them with the models automatically built into crystallographic maps. Finally, we assess the reliability of ADPs refined using *REFMAC* (Murshudov *et al.*, 2011 ▸) as an estimate of the local accuracy of atomic coordinates.

## 2. Materials and methods   

### 2.1. Selection of test-set models and maps   

Structures of proteins and protein–nucleic acid complexes were retrieved from the Protein Data Bank (PDB) together with their target sequences, corresponding cryo-EM maps and half-maps deposited in the Electron Microscopy Data Bank (EMDB; Velankar *et al.*, 2016 ▸) as of 15 April 2019. We selected models with molecular weights below 500 kDa, a reported resolution of better than 4.0 Å and half-maps available for download from the EMDB. We excluded models corresponding to filaments or helical reconstructions, for which a target molecule was not clearly separated in the cryo-EM map.

Initially, all the maps were corrected for over-sharpening, as described below in Section 2.6.2. All models were then refined into the corrected cryo-EM maps using the *ARP*/*wARP* 8.0 module for hybrid real–reciprocal-space model refinement. In the module, a restrained real-space least-squares refinement carried out using the *ARP*/*wARP* utility *loopfit* (as described in Evrard *et al.*, 2007 ▸; Langer *et al.*, 2008 ▸) is followed by 100 *REFMAC* cycles of reciprocal-space refinement using electron scattering factors (Murshudov *et al.*, 2011 ▸). Refinement with *REFMAC* uses additional secondary-structure restraints provided by *ProSMART* (Nicholls *et al.*, 2012 ▸) to complement the information content present in cryo-EM maps. The refinement resulted in an increase of the median of the model-to-map correlation coefficient from 0.79 for deposited maps and models to 0.82 for refined models and corrected maps as obtained using *phenix.map_model_cc* (Afonine *et al.*, 2018 ▸). For further processing, we selected maps and the corresponding refined reference models fulfilling the following criteria.

(i) A real-space correlation coefficient of the reference model refined to the corresponding map as described above of 0.3 or higher.
(ii) A local resolution could be calculated from the deposited half-maps using *ResMap *with default parameters (see Section 2.4 for details; we rejected four maps with EMDB codes EMD-3245, EMD-6455, EMD-6479 and EMD-8559, for which *ResMap* failed to provide results).

A total of 105 proteins and 14 protein–nucleic acid complexes with their maps were selected as the test set. Secondary-structure assignments on a single-residue level calculated for the reference structures using *DSSP* (Kabsch & Sander, 1983 ▸) were downloaded from the RCSB web server (Burley *et al.*, 2019 ▸).

### 2.2. Assessment of the automatically built models   

The models automatically built with *ARP*/*wARP* into the test-set maps were compared with the refined reference structures obtained as described in Section 2.1. For models containing both protein and nucleic acid components, only the protein parts were compared. For model assessment, we used the following definitions.

(i) A residue was *correctly built* if its C^α^ atom was within an arbitrarily selected 2.0 Å distance from a C^α^ atom in the reference structure.
(ii) A residue was *correctly docked* to the sequence if it was correctly built and had the same side-chain type as the corresponding residue in the reference model.
(iii) The *model completeness* is the ratio of correctly built residues to the total number of residues present in the reference model.
(iv) The *sequence coverage* is the ratio of correctly docked residues to the total number of residues present in the reference model.

Analogously, for the final *ARP*/*wARP* models we define correctness of the main chain and sequence assignment as the fraction of the *ARP*/*wARP* model residues that are correctly built and correctly docked, respectively.

### 2.3. Estimation of the accuracy of crystallographic models in a crystallographic test set   

To estimate the accuracy of the models automatically built with *ARP*/*wARP* at a different resolution, we used the crystallographic test set described previously (Chojnowski *et al.*, 2019 ▸). In brief, 400 high-quality crystallographic models and corresponding X-ray diffraction data with a high-resolution limit between 2.0 and 3.0 Å were taken from the PDB-REDO database (Joosten *et al.*, 2014 ▸). The reference models were compared with the models automatically built using *ARP*/*wARP* starting from maps computed from experimental structure-factor amplitudes and model-calculated phases disturbed with an additional 40° uniform phase error. The nearest-neighbour r.m.s.d. was calculated between C^α^ atoms in an *ARP*/*wARP* model and the reference structure (crystallo­graphic symmetry was taken into account). To account for the presence of occasional tracing errors, distances exceeding 2.0 Å were ignored.

### 2.4. Estimation of local resolution in cryo-EM maps   

Local resolution was provided by *ResMap* version 1.1.4 for all half-map pairs in the test set using default parameters and a resolution range from 2.0 to 7.0 Å in 0.5 Å steps. To smooth the local resolution estimates for each residue in the deposited model, we additionally averaged the local resolution values at map grid points within a 1.5 Å distance of the C^α^-atom position and defined these as ‘local resolution at C^α^-atom positions’. The number of models and corresponding C^α^ atoms in each of the ten local resolution ranges are summarized in Table 1 ▸.

### 2.5. Estimation of the accuracy of cryo-EM models   

The nearest-neighbour distances between C^α^ atoms in *ARP*/*wARP* models and those in refined reference structures were computed as a function of local resolution at the reference C^α^-atom positions (defined in Section 2.4). Similarly to the crystallographic models, nearest-neighbour distances greater than 2.0 Å were excluded from the analysis.

### 2.6. Model building into cryo-EM maps with *ARP*/*wARP*   

For all deposited maps, atomic models were built *de novo* and fully automatically using *ARP*/*wARP* 8.0 (command-line script auto_em.sh) using default parameters. The steps of the map-interpretation process are detailed below.

#### 2.6.1. Input map processing   

Deposited cryo-EM maps are usually placed in an artificial ‘box’ much larger than the target molecule, which may affect the performance of model refinement (Nicholls *et al.*, 2018 ▸). Therefore, prior to model building the part of the cryo-EM map corresponding to the target molecule is shifted to the centre of a pseudo-crystallo­graphic unit cell in space group *P*1 with all angles equal to 90°. The required unit-cell dimensions matching the target molecule are estimated using the compact free-atoms model used for a sparse representation of map objects in *ARP*/*wARP* (Morris *et al.*, 2002 ▸). The free-atoms model is built directly into the input map. Subsequently, the input map region corresponding to the free-atoms model is shifted and trimmed with additional 5.0 Å margins.

#### 2.6.2. Correction for map over-sharpening   

Input maps were scaled to adjust the Wilson plot *B* factor of their reciprocal-space intensity falloff, estimated following Popov & Bourenkov (2003 ▸), to a value expected for crystallographic data at the same resolution using the mathematical formulation described by Zwart & Lamzin (2003 ▸).

#### 2.6.3. Overview of the model-building procedure   

The structure-factor amplitudes and phases were calculated from the trimmed, shifted and over-sharpening corrected cryo-EM maps using the *CINVFFT* tool from the Clipper library (Cowtan, 2003 ▸) distributed with *CCP*4 (Winn *et al.*, 2011 ▸). *ARP* */wARP* followed a standard crystallographic protein model-building procedure (Langer *et al.*, 2008 ▸) with a number of specific modifications introduced for the treatment of cryo-EM data as listed below. In the current implementation, the input cryo-EM map is not modified throughout the model-building process. Reciprocal-space model refinement was carried out by *REFMAC* version 5.8.0253 (Murshudov *et al.*, 2011 ▸) using electron scattering factors. The refinement is supplemented with secondary-structure restraints automatically generated using *ProSMART* for each intermediate *ARP*/*wARP* model. Sequence assignment uses an algorithm based on changes of the side-chain density volume at a map threshold (Chojnowski *et al.*, 2019 ▸). Fragmentation of the models is reduced using two independent loop-building algorithms based on a database of short peptides (Chojnowski *et al.*, 2019 ▸) and automatically identified homologous structures (Chojnowski *et al.*, 2020 ▸).

#### 2.6.4. Building a consensus protein model   

The final *ARP*/*wARP* model is constructed from several intermediate models (five by default) using a consensus-modelling approach (Lundström *et al.*, 2001 ▸) in which the intermediate models are combined giving a preference to the most common fragments among them, as described in Chojnowski *et al.* (2020 ▸). This additionally reduces possible tracing errors and helps to construct longer fragments. The resulting consensus model is completed, assigned to the sequence and refined using the standard *ARP*/*wARP* procedure as described in Section 2.6.3.

### 2.7. Implementation and availability   

The benchmarks were performed using the *GNU parallel* software (Tange, 2015 ▸). The developed method has been implemented with the use of the *CCP*4 (Winn *et al.*, 2011 ▸) and *cctbx* (Grosse-Kunstleve *et al.*, 2002 ▸) utilities and libraries. The method was made available in October 2019 as a web server at http://arpwarp.embl-hamburg.de and will be provided for download with the next joint *ARP*/*wARP*–*CCP*4 software release.

## 3. Results   

### 3.1. Overall model-building results   

**Figure 1:**
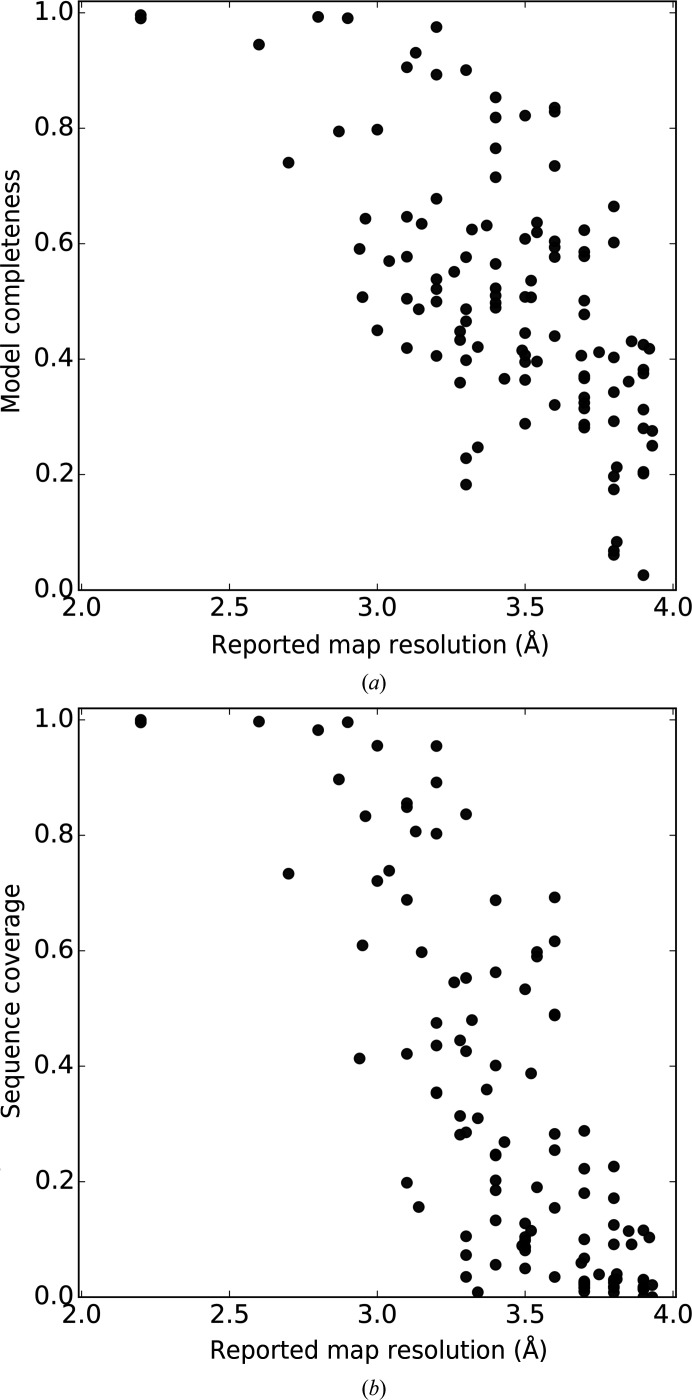
The overall performance of *ARP*/*wARP* model building as a function of reported map resolution: (*a*) fraction of correctly built residues and (*b*) sequence coverage (see Section 2.2 for details).

We observed that the completeness of the *ARP*/*wARP* models built *de novo* into the test-set cryo-EM maps correlates with the reported resolution, although the spread is rather large (Fig. 1 ▸). At a resolution better than 3.0 Å most of the residues can be correctly traced and assigned to the sequence. At lower resolution the completeness of the models is reduced, so that 4.0 Å resolution or a little lower can be regarded as the current limit for correct main-chain tracing and sequence docking, which is consistent with reports for related methods (Terwilliger *et al.*, 2018 ▸).

### 3.2. Local resolution in reference maps   

**Figure 2:**
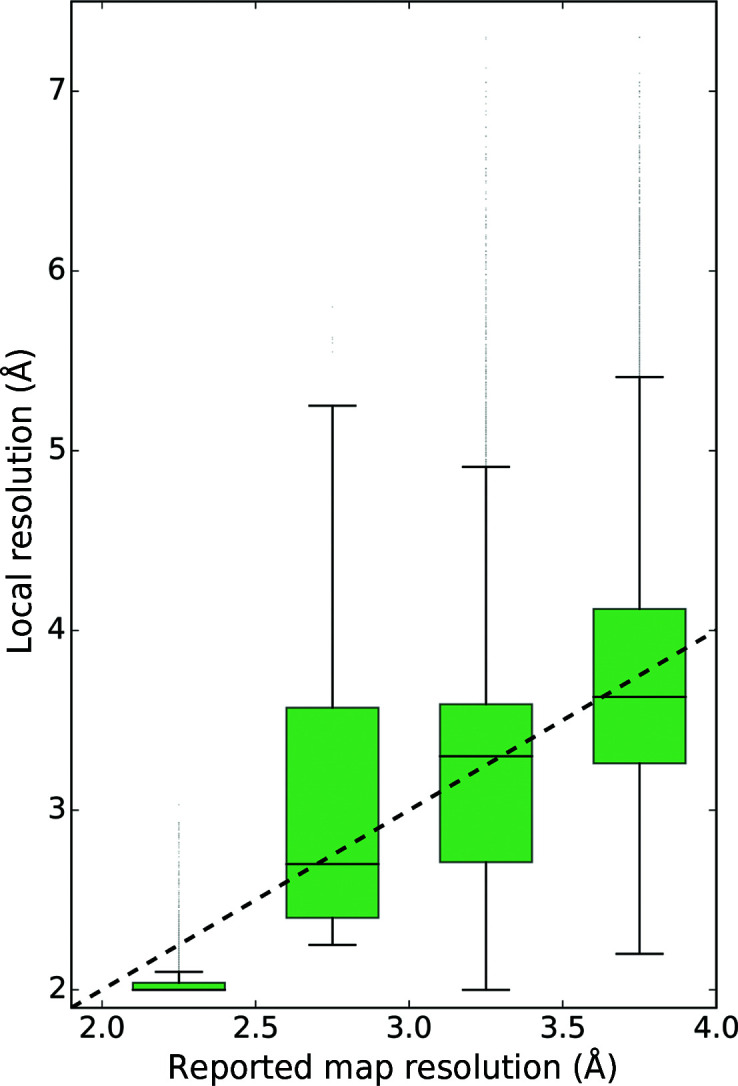
The distribution of local resolution at the C^α^-atom positions in reference models as a function of reported overall resolution of the map. The dashed line is a zero-intercept median-based linear regression model with a slope equal to 1.015 (1), demonstrating that on average half of the residues in deposited models are built into map regions of lower local resolution than reported. The asymmetry of the distributions is seen from the box-plot whiskers, which correspond to the 5th and 95th percentile.

For all reference models a distribution of local map resolution at a single-residue level was obtained as described in Section 2.4. Overall, 50% of residues in the reference models were built into regions with local resolution equal or lower than the reported resolution (Fig. 2 ▸). The distribution of local resolution, however, is asymmetric. We did not observe any differences in local resolution distribution for regions corresponding to α/β parts of the structures compared with loop regions.

### 3.3. Correction for map over-sharpening   

**Figure 3:**
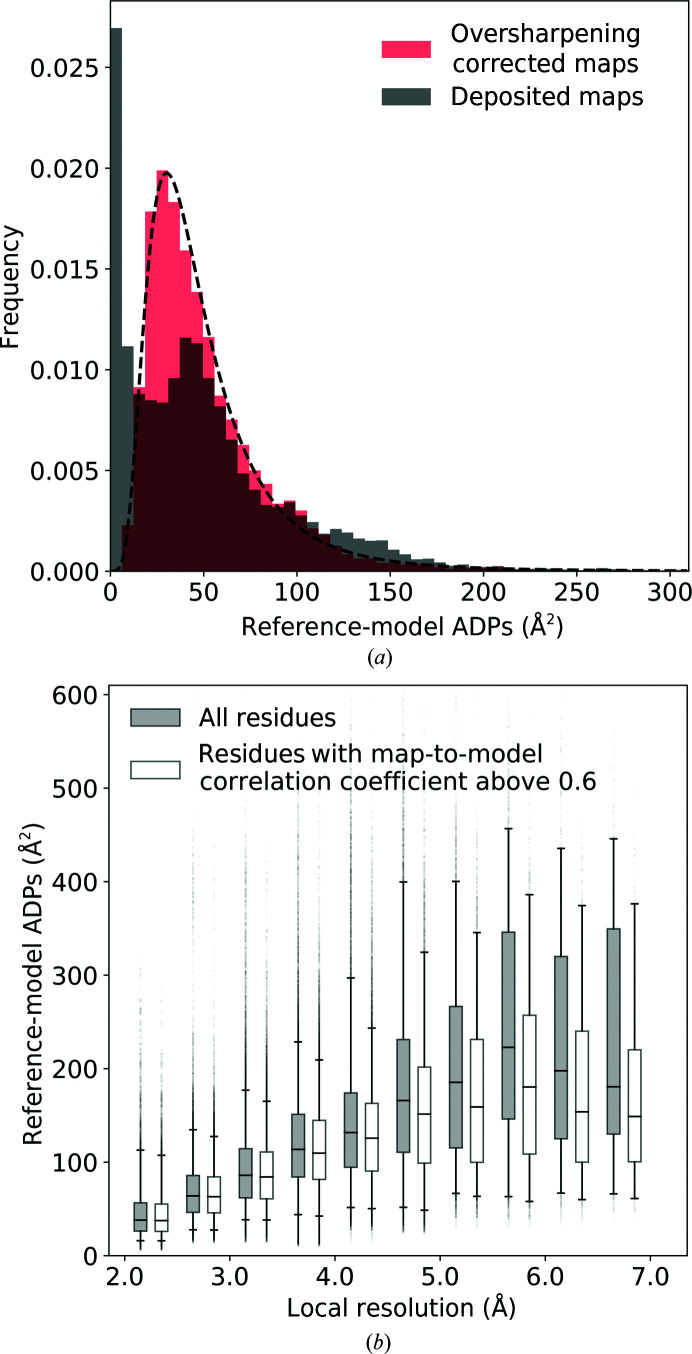
The automated rescaling of cryo-EM maps to account for over-sharpening. (*a*) Distribution of main-chain atomic displacement parameter (ADP) values for the reference models in map regions with local resolution better than 2.5 Å. The dashed curve depicts the inverse-gamma distribution function fitted to the corrected map data. (*b*) ADP values for the main-chain atoms in the reference models for different local resolution regions of corrected cryo-EM maps. The residue-based map-to-model correlation coefficient was calculated using *phenix.map_model_cc*. Box-plot whiskers correspond to the 5th and 95th percentile.

We compared the distributions of main-chain ADP values for the deposited reference models with those refined into maps automatically corrected for over-sharpening. We observed that in the map regions with local resolution better than 2.5 Å the ADP values for many atoms in the deposited models are at the lowest limit of 0.5 Å^2^ (Fig. 3 ▸ *a*). This may be a sign of strong over-sharpening of the map (Masmaliyeva & Murshudov, 2019 ▸). By contrast, the ADP values for the same atoms after refinement into corrected cryo-EM maps fit well to the inverse-gamma distribution function (Fig. 3 ▸ *a*), which was shown to be a good approximation of the distribution of ADP values in refined, good-quality crystal structures (Masmaliyeva & Murshudov, 2019 ▸). We have not observed these effects for map regions with local resolution worse than 2.5 Å.

Generally, for lower local resolution regions of the maps the ADP values of the refined models are higher (Fig. 3 ▸ *b*). We observed that the highest ADP values often correspond to residues which are built either incorrectly or into poorly resolved map regions (Fig. 3 ▸ *b*).

### 3.4. Model completeness   

**Figure 4:**
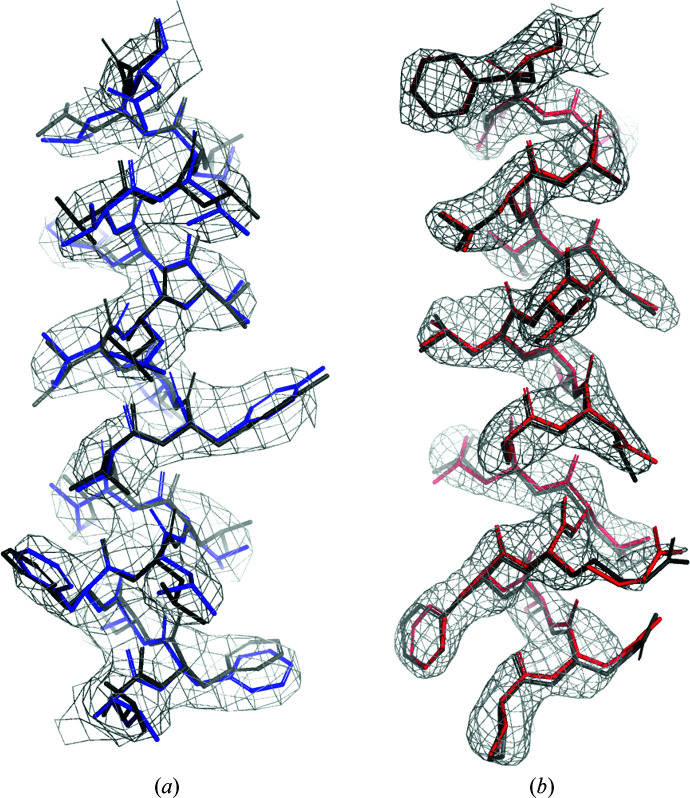
Fragments of models built with *ARP*/*wARP* in map regions with a local resolution of 2.5 Å. (*a*) Human TRV3 at a reported resolution of 3.5 Å. (*b*) Human methemoglobin at a reported resolution of 2.8 Å. The maps are contoured at the author-recommended levels of 3.5σ above the mean for TRPV3 and 9.4σ for methemoglobin. The deposited models are shown in grey.

We observed that the level of detail visible in regions of similar local resolution in different maps is comparable, as exemplified in Fig. 4 ▸. To systematically evaluate this observation we estimated the completeness of the *ARP*/*wARP* models built into map regions of comparable local resolution.

**Figure 5:**
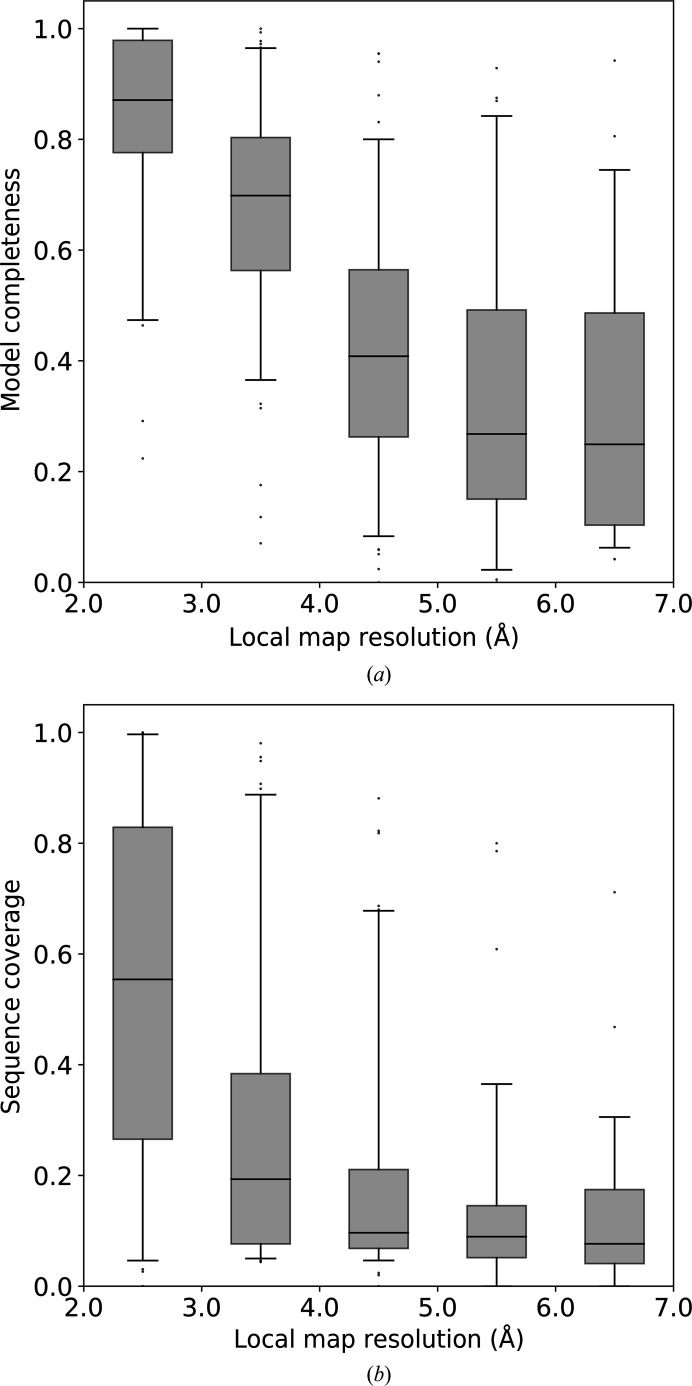
The fraction of reference models that are correctly built by *ARP*/*wARP* in regions of different local map resolution: (*a*) model completeness and (*b*) sequence coverage. Box-plot whiskers correspond to the 5th and 95th percentile.

We found that the model completeness strongly correlates with local resolution, and that the median model completeness drops from 90% for local resolution better than 3.0 Å to below 30% for local resolution worse than 5.0 Å (Fig. 5 ▸ *a*). Similarly, sequence coverage reduces from over 50% for local resolution better than 3.0 Å to about 10% for resolution worse than 5.0 Å (Fig. 5 ▸ *b*). It is noted, however, that the spread of sequence coverage is large. This may be attributed to the fact that in this work the local resolution is linked to individual C^α^ atoms, while sequence coverage depends on the spatial proximity of approximately equal local resolution regions allowing a sequence fragment to be correctly docked. We did not observe any differences in model completeness between map regions corresponding to secondary-structure elements and loops.

To evaluate the predictive power of local resolution to determine *ARP*/*wARP *model completeness, we built logistic regression models using local and FSC-based resolution estimates as independent variables. We observed that the adjusted proportion of correct predictions (Hoetker, 2007 ▸) was larger for the model with local resolution as an independent variable [0.133 (3) and 0.013 (7), respectively]. This demonstrates that local resolution is a stronger determinant of model completeness. The standard errors of the adjusted proportion of correct predictions, given in parentheses, are bootstrap approximations.

### 3.5. Model correctness   

**Figure 6:**
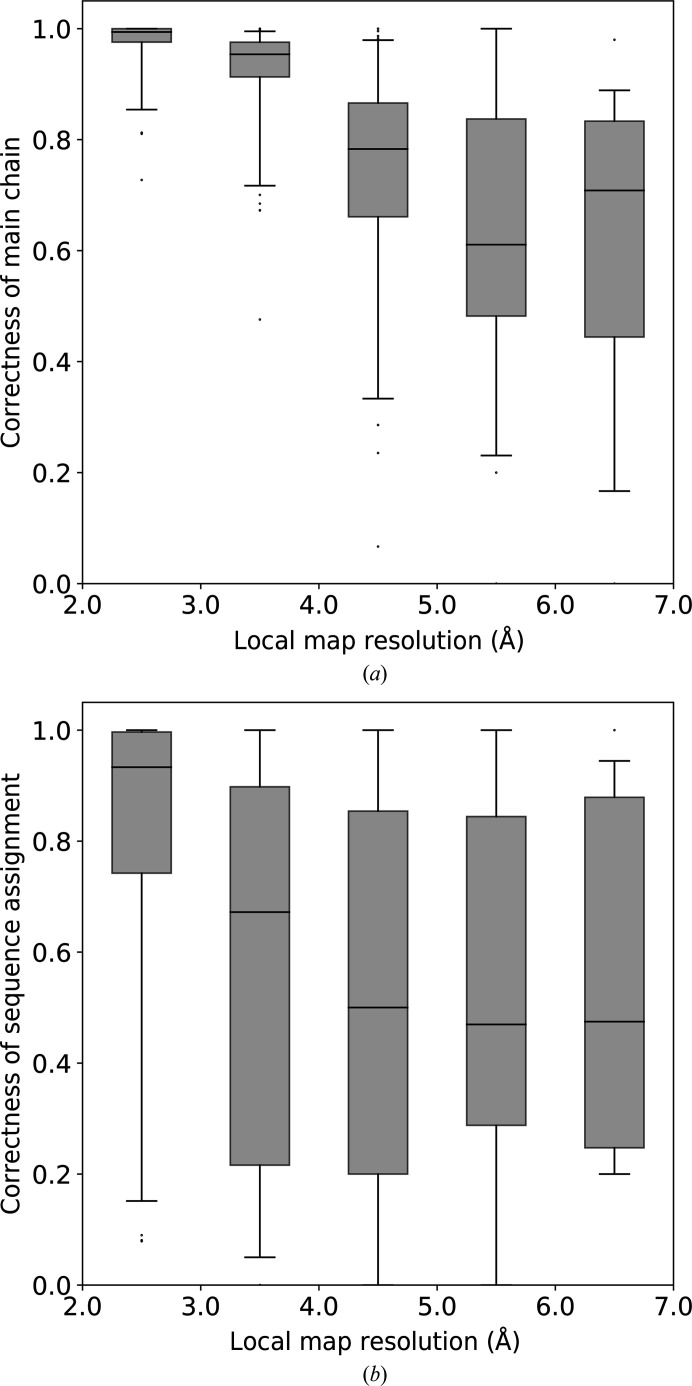
The fraction of *ARP*/*wARP* models that are correctly built in regions of different local resolution: (*a*) main-chain traces and (*b*) sequence assignment. Box-plot whiskers correspond to the 5th and 95th percentile.

We have evaluated *ARP*/*wARP* model correctness, defined as the fraction of residues that are correctly built and also correctly assigned to the sequence, in cryo-EM map regions of comparable local resolution. It was observed that the median correctness of the main chain is over 90% for map regions with local resolution better than 4.0 Å and is above 70% at local resolutions between 4.0 and 7.0 Å (Fig. 6 ▸ *a*). In addition, the mean fraction of residues correctly docked into the sequence exceeds 90% at a resolution better than 3.0 Å and is around 60–70% at local resolutions between 3.0 and 7.0 Å. The correctness of sequence assignment is spread more broadly than the correctness of the main chain, as can be seen in the box plots (Figs. 6 ▸ *a* and 6 ▸ *b*). We did not observe any differences in model correctness between regions corresponding to α/β parts of the structures and loop regions.

Similarly to the model completeness, we built logistic regression models for model correctness using local resolution and FSC-based resolution estimates. We did not, however, observe any statistically significant difference between the adjusted proportions of correct model predictions.

### 3.6. Accuracy of atomic coordinates   

**Figure 7:**
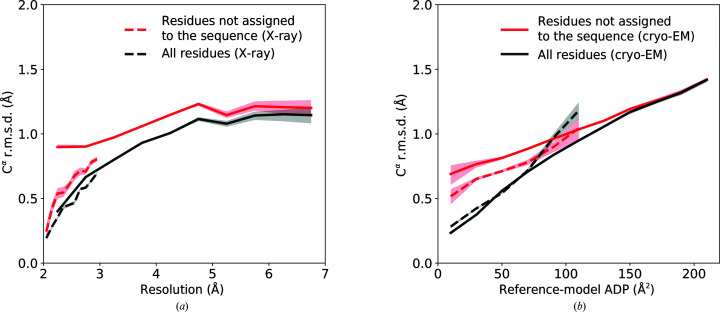
R.m.s.d. of correctly built *ARP*/*wARP* model C^α^ positions from those in the refined reference models as a function of (*a*) local cryo-EM map or overall X-ray data resolution and (*b*) mean ADP of the main-chain atoms in refined reference models. The solid and dashed lines correspond to cryo-EM and X-­ray models, respectively. The shaded areas indicate bootstrap approximations of the 90% confidence interval.

We evaluated the deviation of C^α ^atoms in the *ARP*/*wARP* models from those in the refined coordinates of deposited models in the test set. The r.m.s.d. values were calculated for local resolutions between 2.0 and 7.0 Å in ten bins (each with a width of 0.5 Å) for all of the residues in the test-set models. We regard the deviation of C^α^-atom coordinates as an estimate of the coordinate error. These estimates correlated with the local resolution, particularly within the range 2.0–5.0 Å (Fig. 7 ▸ *a*). The coordinate-error estimates obtained using a similar approach for crystal structure models at a resolution better than 3.0 Å are comparable (Fig. 7 ▸ *a*).

We also compared the r.m.s.d. in C^α^-atom positions with their refined ADP values (Fig. 7 ▸ *b*). We conclude that the coordinate errors for cryo-EM and crystallographic models are very similar for ADP values of up to about 80 Å^2^ (Fig. 3 ▸ *b*).

The coordinate error is significantly lower for residues that could be assigned to the sequence. Indeed, protein fragments built with *ARP*/*wARP* and not assigned to the target sequence, modelled as polyglycine chains, have a coordinate error almost twice as high compared with residues with the side chains assigned (Fig. 7 ▸). This may be related to the fact that about half the number of stereochemical restraints can be applied to the refinement of models without assigned side chains. It is worth noting that polyglycine model fragments built into high local resolution regions (around 2.0 Å) of cryo-EM maps have a significantly higher coordinate error compared with crystallo­graphic models (Fig. 7 ▸ *a*). The fraction of polyglycine fragments in these map regions, however, is relatively small (9% at a local resolution better than 2.5 Å).

## 4. Discussion and conclusions   

In this work, we have systematically evaluated the reliability of models automatically built into cryo-EM maps using *ARP*/*wARP*. We demonstrated that the local resolution of the cryo-EM map is a suitable parameter for determining the accuracy and completeness of the model and is superior to the Fourier shell correlation-based resolution estimate. We also observed that large fractions of the deposited cryo-EM maps and models correspond to regions with a local resolution that may be too low for automated interpretation using currently available model-building approaches. This may be a reason for the rapid decrease with reported resolution in the completeness of automatically built models using *ARP*/*wARP* as well as other methods (Mendez & Stagg, 2018 ▸; Terwilliger *et al.*, 2018 ▸). We observed no significant difference in the completeness and correctness of *ARP*/*wARP* models for α/β protein fragments, even though the secondary-structural elements are regarded as better resolved in lower resolution maps compared with loop regions.

We observed that the deviations between models built *de novo* using *ARP*/*wARP* and the deposited models depend on the local resolution. These deviations are comparable to those for *ARP*/*wARP* crystallographic models at similar crystallo­graphic resolution. Overall, the accuracy of the models built into cryo-EM maps is, on average, sufficiently high, even at a resolution worse than 4.0 Å. We also note that the accuracy of *ARP*/*wARP* models is better for chain fragments that were assigned to the sequence. Further studies may be required to clarify whether sequence-assignment algorithms are sensitive to the accuracy of the main chain in the first place or whether the presence of side chains results in more accurate refinement of the structure (Herzik *et al.*, 2019 ▸).

In this work, we used deposited cryo-EM models which were re-refined and used as a reference, although they may have contained some errors (Afonine *et al.*, 2018 ▸). However, the large size of the test set used provided a sufficiently clear overall picture. More sophisticated estimates of the accuracy of models built into cryo-EM maps could be obtained with a methodology, already employed in crystallography, in which the same structures at a comparable resolution are built, refined, validated and deposited independently by different groups (Daopin *et al.*, 1994 ▸). We believe that the results presented in this work may serve as a firm foundation for such a study in the future.

Several studies have reported that the ADPs in deposited models obtained from cryo-EM maps may be insufficiently informative (Wlodawer *et al.*, 2017 ▸; Herzik *et al.*, 2019 ▸). We demonstrated that this could be related to excessive over-sharpening of cryo-EM maps. We showed that for models re-refined against cryo-EM maps corrected for over-sharpening, the atomic ADP values correlate with the accuracy of protein backbone atoms. Moreover, the dependence is similar to that for crystal structures (Daopin *et al.*, 1994 ▸). Overall, refinement of cryo-EM models into maps with their Wilson plot *B* factor scaled to a value expected for crystallographic data at a similar resolution proved very efficient. It must be stressed, however, that in this work we used the ADPs of re-refined and complete deposited models. The ADP values of models that are incomplete or not fully refined may be less reliable (Masmaliyeva & Murshudov, 2019 ▸).

Map sharpening in cryo-EM is beneficial for manual map interpretation, helping to emphasize features such as side-chain conformations (Nicholls *et al.*, 2018 ▸). It has also been noted that excessive map sharpening or blurring may result in unstable refinement and lead to physically unreasonable values and distributions of ADPs (Masmaliyeva & Murshudov, 2019 ▸). It would be useful if deposited cryo-EM maps were to have their reciprocal-space intensity falloff adjusted following commonly accepted rules, which would ease the comparison of independently determined models. The simple automated approach to over-sharpening correction presented in this work could be of assistance in preparing cryo-EM maps for model refinement and deposition.

In the work presented here, we demonstrated that *ARP*/*wARP* models can be used as a simple means of assessing cryo-EM map interpretability, for example when multiple reconstructions are available for a single target. In such cases the *ARP*/*wARP* models built *de novo* into cryo-EM maps are reliable and the completeness of the models correlates with the local resolution of the corresponding map regions. Therefore, maps for which *ARP*/*wARP* can build the largest fraction of the target structure should in general be easier to interpret and the corresponding automatically built models should be easier to complete manually. From this perspective, it is important to mention that *ARP*/*wARP* requires minimal user input and is relatively fast. The interpretation of all 119 cryo-EM maps used in this study took 1461 h of a single CPU core: 12 h per map on average. This is about three orders of magnitude faster than the time required for tracing models using *Rosetta* in the analysis of representative segments in 57 cryo-EM maps in a previous study (Mendez & Stagg, 2018 ▸).

## Figures and Tables

**Table 1:** Number of models and C^α^ atoms in the ten local resolution ranges used in this work

	Local resolution range (Å)									
	2.0–2.5	2.5–3.0	3.0–3.5	3.5–4.0	4.0–4.5	4.5–5.0	5.0–5.5	5.5–6.0	6.0–6.5	6.5–7.0
C^α^ atoms	18349	19919	33157	22087	12862	3649	1544	591	341	230
Models	30	52	86	90	84	60	43	35	23	18
